# *Hormographiella aspergillata*: an emerging basidiomycete in the clinical setting? A case report and literature review

**DOI:** 10.1186/s12879-020-05679-z

**Published:** 2020-12-11

**Authors:** Maxime Moniot, Rose-Anne Lavergne, Thomas Morel, Romain Guieze, Florent Morio, Philippe Poirier, Céline Nourrisson

**Affiliations:** 1 Laboratoire de Parasitologie-Mycologie, CHU Clermont-Ferrand, CHU Gabriel Montpied, 58 rue Montalembert, 3IHP, 63003 Clermont-Ferrand Cedex 1, France; 2 Equipe Interactions Hôte-Parasite, Laboratoire Microorganismes : Génome et Environnement, CNRS, Université Clermont-Auvergne, Clermont-Ferrand, France; 3 Laboratoire de Parasitologie-Mycologie, Département de Mycologie Médicale, Hôpitaux Universitaires de Nantes, Universités Nantes Atlantique, EA1155-IICiMed, Institut de Recherche en Santé 2, Nantes, France; 4 Service d’Hématologie Clinique, CHU Clermont-Ferrand, Clermont-Ferrand, France

**Keywords:** *Hormographiella aspergillata*, *Coprinus cinereus*, Mould, Antifungal susceptibility, Fungal colonization, Basidiomycete

## Abstract

**Background:**

Filamentous basidiomycetes are mainly considered to be respiratory tract colonizers but the clinical significance of their isolation in a specimen is debatable. *Hormographiella aspergillata* was first reported as a human pathogen in 1971. We discuss the role of this mold as a pathogen or colonizer and give an update on diagnostic tools and in vitro antifungal susceptibility.

**Case presentation:**

We identified three cases of *H. aspergillata* with respiratory symptoms in a short period of time. One invasive infection and two colonizations were diagnosed. Culture supernatants showed that *H. aspergillata* can produce galactomannan and β-D-glucan but not glucuronoxylomannan. For the first time, isavuconazole susceptibility was determined and high minimum inhibitory concentrations (MICs) were found. Liposomal amphotericin B and voriconazole have the lowest MICs.

**Conclusion:**

To date, 22 invasive infections involving *H. aspergillata* have been reported. On isolation of *H. aspergillata,* its pathogenic potential in clinical settings can be tricky. Molecular identification and antifungal susceptibility testing are essential considering high resistance against several antifungal therapies.

## Background

Filamentous basidiomycetes are mainly considered to be respiratory tract colonizers but increasingly these molds are being documented in invasive infections [1]. Hence, the clinical significance of their isolation in a specimen is debatable. *Hormographiella aspergillata* is a filamentous basidiomycete growing on horse dung. It was found in numerous environmental substrates and first reported as a human pathogen in 1971 [2–4]. Since, a few infections were reported all over the world with various clinical outcomes, essentially pulmonary but also disseminated or located to the eye or the skin [2, 5–22]. Thus, data are sparse for the diagnosis and management of such infections. Here, we report a new case of human infection involving *H. aspergillata* and two cases of colonization. We then review all previously published cases and discuss diagnostic strategy and clinical management.

## Case presentation

**Fig. 1:**
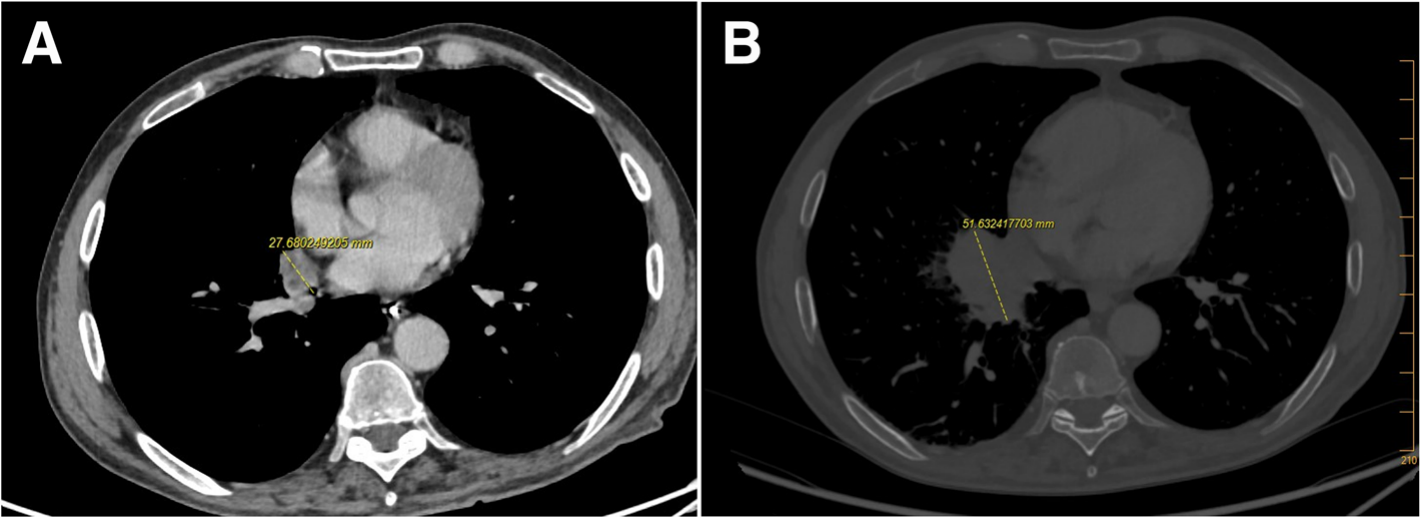
Chest computed tomography scan of HA1 patient showing **a** right lower lobe opacification and **b** increase in the lesion size 1 week later

**Fig. 2:**
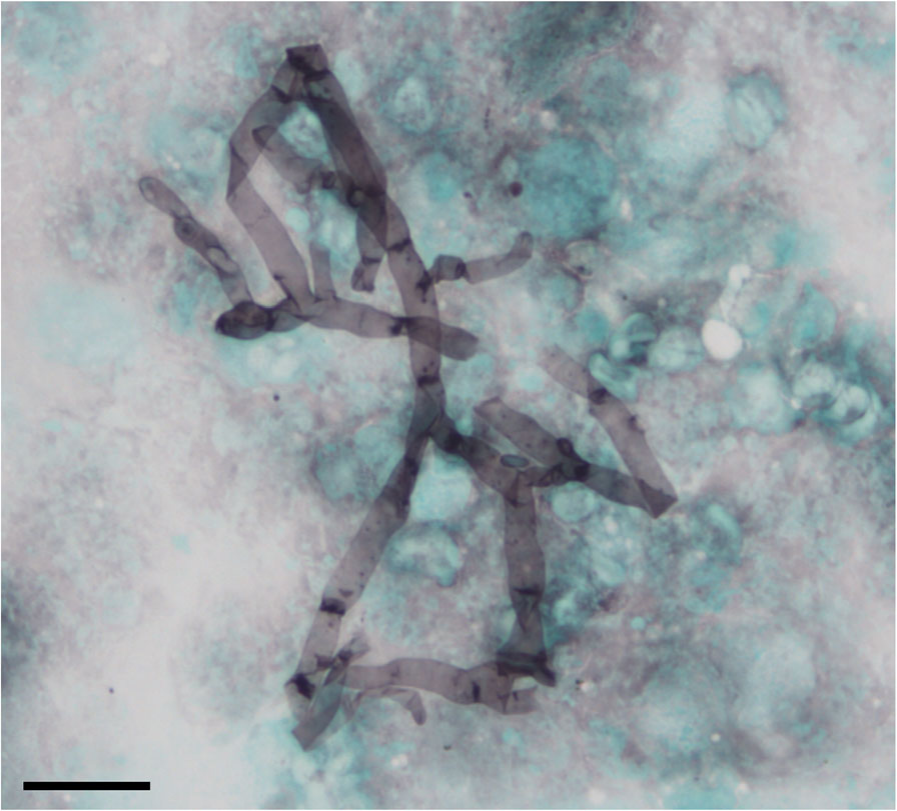
Microscopy examination of bronchoalveolar lavage from patient HA1 by Gomori-Grocott staining showing the presence of septate hyphae. Scale-bar: 10 μm

The first case (HA1) was an 70-year-old man admitted to the hematology department for prolonged febrile neutropenia and anorexia. He had a history of acute myeloid leukemia (AML) and hematopoietic stem cell transplantation (HSCT). His C-reactive protein (CRP, positivity threshold value: 3 mg/L) was 135 mg/L and empirical antibiotic therapy (ceftriaxone) was started at day 210 (D210, 7th month) post-HSCT. Chest computed tomography (CT) scan showed right lower lobe opacification (Fig. 1a) that had increased 1 week later (Fig. 1b). Invasive fungal infection (IFI) was suspected, and liposomal amphotericin B (lAmB 5 mg/kg/day) was started on D232 (7th month). Microscopic examination of a bronchoalveolar lavage (BAL) sampled at D237 (7th month) showed septate hyphae (Fig. 2) but cultures on Sabouraud media incubated at 25 °C and 35 °C were sterile after 7 days. *H. aspergillata* was identified by sequencing the internal transcribed spacer (ITS) region of fungi directly from the BAL. Interestingly, serum galactomannan monitoring was negative (< 0.1 on repeated samples; Platelia® *Aspergillus* assay, Bio-Rad; positivity threshold index: > 0.5) and β-D-glucan (Fungitell®, Cape Cod; positivity threshold value: 80 pg/mL) was weakly positive on D237 (7th month; 98 pg/mL) but negative on D248 (8th month; 46 pg/mL). In accordance with the 2008 European Organization for Research and Treatment of Cancer/Invasive Fungal Infections Cooperative Group and the National Institute of Allergy and Infectious Diseases Mycoses Study Group (EORTC/MSG) criteria, the patient was classified as having probable IFI [23]. His condition worsened following pulmonary *Stenotrophomonas maltophilia* infection and so it was decided to initiate palliative care. lAmB was stopped on D253 (8th month), 3 weeks after its introduction. The patient died on D298 (9th month).

**Fig. 3:**
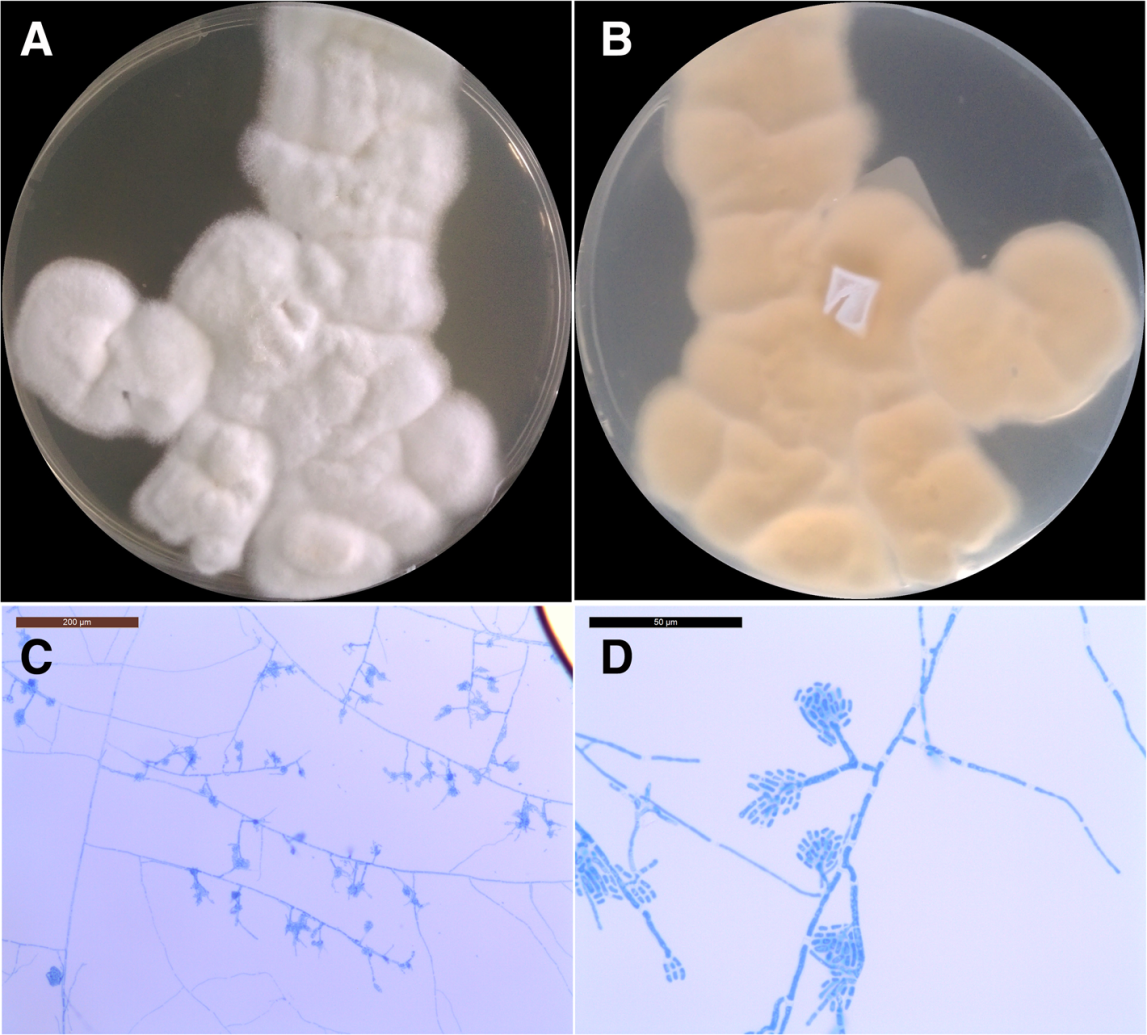
Macroscopic and microscopic morphology of *Hormographiella aspergillata* on potato dextrose agar (PDA) subculture after 3 days of incubation at 25 °C. **a** White to cream colored velvety colonies with irregular margin on the recto side. **b** Verso side of the colonies showing light yellow color. **c**, **d** Slide culture of *Hormographiella aspergillata* showing hyaline septate hyphae with conidiophores and cylindrical arthroconidia without clamp connection, scale-bar: 200 μm (**c**) and 50 μm (**d**)

The second patient (HA2) was a 49-year-old man admitted to the intensive care unit for pneumopathy with acute respiratory failure. He had a history of psychiatric disorders, diabetes mellitus, asthma, smoking and middle cerebral artery stroke with persistent sequelae. CRP was negative on admission. The following day, it was positive at 108.0 mg/L but procalcitonin remained negative. Mechanical ventilation and empirical antibiotic therapy (ceftazidime) were initiated. A mucous plug containing purulent secretions in the left lung was removed by fibroscopy and transmitted to Bacteriology and Mycology Laboratories. Microscopy examinations of samples were negative but cultures identified oropharyngeal microbiota associated with a white mold on Sabouraud media at 25 °C and 35 °C after 7 days. Subcultures of mold grew with white to slightly cream-colored velvety colonies (Fig. 3a and b) on potato dextrose agar media. Microscopy examination of cultures showed hyaline septate hyphae with conidiophores producing cylindrical arthroconidia (Fig. 3c and d). *H. aspergillata* identification was confirmed by sequencing the ITS region. In vitro antifungal susceptibility testing was performed via broth microdilution technique according to the European Committee on Antimicrobial Susceptibility Testing (EUCAST) guidelines [24]. Minimum inhibitory concentrations (MICs) are given in Table 1. The chest CT scan was unremarkable and there was no risk factor for IFI and so no antifungal therapy was initiated. The inflammatory syndrome decreased rapidly 3 days later, and the patient’s condition improved. A putative diagnosis of bacterial aspiration pneumonia with fungal colonization was established.

**Table 1:** Antifungal susceptibility testing of *Hormographiella aspergillata* from the literature and our cases

			MIC (mg/L) for single isolates								
References	Year	Method	AmB	5-FC	FCZ	ITZ	VRZ	PSZ	ISA	CSF	MCF
Speller and MacIver [2].	1971	Dilution method on Yeast Morphology Agar	0.25	> 250	**/**	**/**	**/**	**/**	**/**	**/**	**/**
Verweij et al. [6]	1997	Broth macrodilution method with RPMI-1640	0.5	**/**	**>** 64	8	**/**	**/**	**/**	**/**	**/**
Lagrou et al. [8]	2005	E-test®	0.5	**/**	**/**	2	0.25	**/**	**/**	**/**	**/**
Abuali et al. [10]	2009	Broth microdilution method according to CLSI M38-A2	4	**/**	**/**	**/**	0.25	0.5	**/**	**>** 2	> 4
Conen et al. [11]	2011	E-test®	0.5	**/**	256	/	0.125	**/**	**/**	32	/
Conen et al. [11]	2011	E-test®	0.5	**/**	**/**	**/**	0.125	2	**/**	**/**	**/**
Conen et al. [11]	2011	E-test®	0.5	/	> 256	/	0.25	**/**	**/**	> 32	/
Suarez et al. [12]	2011	Broth microdilution method according to EUCAST	2	> 64	64	> 8	1	**/**	**/**	2	/
Bojic et al. [14]	2013	E-test®	0.094	> 32	> 256	/	0.125	0.064	**/**	**/**	/
Nanno et al. [17]	2016	Not available	0.125	> 64	4	0.25	0.015	/	**/**	**/**	> 16
Koncan et al. [18]	2016	Sensititre YeastOne	0.12	/	16	/	0.03	0.125	**/**	**/**	/
Jain et al. [21]	2019	Not available	0.3	**/**	**/**	4	0.5	**/**	**/**	4	4
Our report HA2	2019	Broth microdilution method according to EUCAST	0.125	**/**	**/**	> 8	2	4	4	**/**	**/**
Our report HA3	2019	Broth microdilution method according to EUCAST	0.125	**/**	**/**	> 8	8	> 8	16	**/**	**/**

*AMB Amphotericin B, *5-FC* Flucytosine, *FCZ* Fluconazole, *ITZ* Itraconazole, *VRZ* Voriconazole, *PSZ* Posaconazole, *ISA* Isavuconazole. *CSF* Caspofungin, *MCF* Micafungin

The third patient (HA3) was a 28-year-old woman admitted for investigation of an inflammatory disease affecting the central nervous system treated by methylprednisolone for 3 days (1 g/day). Bronchial fibroscopy was performed along with other investigations. Initial microscopy examination of the sample was negative but *H. aspergillata* grew after 3 weeks on Lowenstein-Jensen medium at 35 °C because of mycobacterial suspicion (identification confirmed by ITS sequencing). Antifungal susceptibility testing was performed as described above (Table 1). The patient was asymptomatic and her chest CT scan normal, suggesting colonization, and so no antifungal treatment was initiated.

## Literature review

We reviewed the literature since 1971 to date using the terms “*Hormographiella aspergillata*” or “*Coprinus cinereus”* and “infection” in MEDLINE database (Tables 1 and 2). For each strain, antifungals MIC with the method used were reported in Table 1 when available. According to the 2008 EORTC/MSG criteria, all probable or proven IFI due to *H. aspergillata* were reported in Table 2 with significant clinical details.

**Table 2:** Literature review of *Hormographiella aspergillata* infections in humans published since 1971

References	Country	Year	Infection site	EORTC/MSG classification	Underlying disease	Diagnosis Samples - Methods		Antifungal treatment	Surgery	Outcome
Speller and MacIver [2].	England	1971	Heart	Proven	Prosthetic Valve	Autopsy	Histology + culture	None	Yes	Died
Nenoff et al. [5]	Germany	1997	Lung	Proven	ALL	Autopsy	Histology + culture	AmB	No	Died
Verweij et al. [6]	Netherlands	1997	Lung	Proven	ALL	Autopsy	Histology + culture + RFLP	AmB ➔ ITZ	No	Died
Surmont et al. [7]	Belgium	2002	Lung	Proven	Lymphoma	Transthoracic puncture	DE + culture	AmB	No	Alive
Lagrou et al. [8]	Belgium	2005	Lung	Probable	AML	BAL	DE + culture	CSF	No	Died
Greer et al. [9]	USA	2008	Heart	Proven	Valve prosthesis	Resected valve	Histology + culture	lAmB	Yes	Alive
Abuali et al. [10]	USA	2009	Skin	Proven	AML	Skin biopsy	Culture	VRZ ➔ PSZ + CSF ➔ lAmB + CSF	No	Died
Conen et al. [11]	Switzerland	2011	Lung, eye, CNS, blood	Proven	AML	Autopsy	Histology + culture	VRZ ➔ PSZ ➔ CSF	No	Died
			Lung	Proven	AML	Lung biopsy	Histology + culture	VRZ ➔ PSZ ➔ lAmB ➔ VRZ	Yes	Died
			Lung	Proven	AML	Lung biopsy	Histology + culture	VRZ ➔ lAmB ➔ VRZ	Yes	Died
Suarez et al. [12]	France	2011	Lung	Proven	BAL	Lung biopsy	DE + culture	CSF ➔ VRZ ➔ lAmB	No	Alive
			Lung	Proven	X-ALD	Autopsy	Histology + culture + PF-PCR	CSF ➔ lAmB	No	Died
Pang et al. [13]	France	2012	Lung	Proven	ALL	Lung biopsy	Culture	CSF ➔ VRZ ➔ lAmB	No	Alive
Bojic et al. [14]	Austria	2013	Skin, lung	Proven	AML	Skin biopsy	Histology	CSF ➔ lAmB + VRZ	Yes	Died
Corzo-León et al. [15]	USA	2015	Lung	Probable	AML	BAL	Culture	VRZ ➔ lAmB	No	Died
Heiblig et al. [16]	France	2015	Sinus, orbit, CNS	Proven	AML	Sinus biopsy	DE + culture	CSF ➔ PSZ ➔ lAmB + VRZ	Yes	Died
Nanno et al. [17]	Japan	2016	Lung, CNS, small intestine	Proven	MDS	Lung biopsy	Histology + culture + βDG	ITZ ➔ lAmB + CSF ➔ VRZ + MCF ➔ VRZ + lAmB	No	Died
Koncan et al. [18]	Italy	2016	Lung	Proven	MPAL	Lung resection	Culture + PF-PCR + βDG	PSZ ➔ VRZ	Yes	Alive
Correa-Martinez et al. [19]	Germany	2017	Skin	Proven	Nephroblastoma	Skin biopsy	Histology + culture	PSZ	Yes	Alive
Godet et al. [20]	France	2017	Lung	Proven	AML	Lung biopsy	DE + PF-PCR	VRZ ➔ lAmB (IV + nebulized)	Yes	Alive
Jain et al. [21]	India	2019	Eye	Proven	Intraocular lens implantation	Corneal tissue	DE + culture + PF-PCR	Natamycin + ITZ ➔ VRZ	Yes	Alive (loss of the eye)
Chauhan et al. [22]	USA	2019	Lung, CNS	Proven	CML	Autopsy	Histology + culture + PF-PCR + βDG	MCF	No	Died
Our report HA1	France	2019	Lung	Probable	AML	BAL	DE + PF-PCR + βDG	lAmB	No	Died

Search for previously published cases using the terms “*Hormographiella aspergillata*” or “*Coprinus cinereus* infection” in MEDLINE database

*ALL Acute lymphoid leukemia, *AML* Acute myeloid leukemia, *BAL* Biphenotypic acute leukemia, *X-ALD* X-linked adrenoleukodystrophy, *CML* Chronic myeloid leukemia, *MDS* Myelodysplasia syndrome, *MPAL* Mixed phenotype acute leukemia, *CNS* Central nervous system, *AmB* Deoxycholate amphotericin B, *ITZ* Itraconazole, *CSF* Caspofungin, *VRZ* Voriconazole, *PSZ* Posaconazole, lAmB Liposomal amphotericin B, *MCF* Micafungin, *IV* Intravenous, *RFLP* Restriction fragment length polymorphism, *DE* Direct examination, *PF-PCR* Pan-fungal-polymerization chain reaction, *βDG* 1, 3-beta-D glucan

## Discussion and conclusions

*Hormographiella aspergillata* is an environmental filamentous basidiomycete found in numerous substrates including soils, leaves, pressmud compost and in the air [3, 4]. It is the anamorph form of *Coprinopsis cinerea* (formerly *Coprinus cinereus*), which commonly grows on horse dung. It can be an opportunistic pathogen and is the second filamentous basidiomycete responsible for human infection after *Schizophyllum commune* [25]. To date, 22 invasive infections involving *H. aspergillata* have been reported (Table 2), mostly identified by sequencing of the 28S rDNA or ITS regions [2, 5–22]. Most cases were diagnosed in Europe, but some were documented in the United States, Japan and India, in both rural and urban areas [2, 5–22]. Infection cases occurred mainly in neutropenic patients. Although *H. aspergillata* is primarily responsible for pulmonary infections it can occasionally cause primary cutaneous lesions [10, 14, 19]. *H. aspergillata* is able to grow in blood cultures [11] and a few cases of disseminated infections have been reported, affecting the small intestine, the eye and the brain [11, 16, 17, 22]. Interestingly, three cases of IFI have also been reported in immunocompetent patients following cardiac or ophthalmic surgery [2, 9, 21]. The most contributive samples were biopsies, but some cases were diagnosed with BAL. [8, 15] *H. aspergillata* grows well on different fungal media without cycloheximide at 25 °C or 35 °C. However, diagnosis can be challenging in patients with negative cultures, as for the HA1 patient, whose strain was probably inhibited by the concomitant antifungal treatment. To date, there are insufficient data to draw any conclusions about biomarkers since in all documented reports galactomannan assays were negative and only two observations reported strongly positive β-D-glucan antigens greater than 500 pg/mL [18, 22]. We attempted to evaluate the production of galactomannan, β-D-glucan and glucuronoxylomannan antigens on in vitro cultures. Glucuronoxylomannan is a capsular antigen of *Cryptococcus neoformans* widely used to diagnose cryptococcosis. Some cross-reactions have already been described with other basidiomycete pathogens such as *Trichosporon* sp. or even *Coprinopsis cinerea* [26]. Interestingly, culture supernatants from strains HA2 and HA3 showed that *H. aspergillata* can produce galactomannan and β-D-glucan but not glucuronoxylomannan (Table 3). Although, as for HA1, results in sera are variable, biomarker assays could provide supplementary evidence in patients with suspected IFI.

**Table 3:** Galactomannan (GM), β-D-glucan and glucuronoxylomannan antigen assays on culture supernatant. For each strain, 5 to 10 colonies incubated at 35 °C for 4 days on Sabouraud media were suspended in 1 ml distilled water. After vigorous agitation, the suspensions were centrifuged for 5 min at 10,000 g. 1, 1:10 and 1:100 dilutions of the supernatants were then tested with Platelia® *Aspergillus* assay (Bio-Rad, France), Fungitell® assay (Associates of Cape Cod Inc., USA) and Biosynex® CryptoPS assay (Biosynex, France) according to the manufacturer’s recommendations

Isolate	Dilution factor	Galactomannan	β-D-glucan (pg/mL)	Glucuronoxylomannan
HA2	1	> 3,5	> 500	Negative
	10	> 3,5	> 500	n.d.
	100	0,2423	51,048	n.d.
HA3	1	> 3,5	> 500	Negative
	10	> 3,5	> 500	n.d.
	100	0,4197	98,804	n.d.

*n.d.* Not determined

*H. aspergillata* can also be a colonizer of the respiratory tract, as illustrated in our three patients, all of whom had an underlying respiratory condition. The weak clinical significance of the isolation of basidiomycetes in healthy subjects, in contrast with their life-threatening potential in immunocompromised patients, has already been described with *Schizophyllum commune* or *Ceriporia lacerata*, for example [27, 28]. These fungi are widely present in the environment, and their spores are easily inhaled and can grow in pulmonary alveoli in cases of local or systemic impaired function of alveolar macrophages.

As yet there are no EUCAST nor Clinical and Laboratory Standards Institute (CLSI) breakpoints to interpret the antifungal MICs for *H. aspergillata*. However, previous articles have reported in vitro resistance to echinocandins, fluconazole along with high MIC for flucytosine (Table 1). We found higher MICs for isavuconazole (4 and 16 mg/L) than what is usually observed for basidiomycetes [28, 29]. In the light of our findings and data from the literature, lAmB and voriconazole have the lowest MICs. However, *H. aspergillata* infections have a poor prognosis even when surgical debridement is performed.

In conclusion, on isolation of *H. aspergillata,* its pathogenic potential in clinical samples should be interpreted together with the patient’s history. Formal identification of the fungus can be tricky and usually requires molecular tools in addition to culture. Basidiomycetes can also be contaminants or colonizers and so microscopy examination of samples and/or histology in combination with biomarkers are crucial for diagnosis. Respiratory tract colonization is probably not uncommon given that the fungus is widespread in the environment but seems to be restricted to patients with underlying respiratory diseases. lAmB and voriconazole seem to be the antifungals of choice.

## Data Availability

New genome sequences obtained in this study have been deposited in GenBank under accession numbers MN841917, MN841918 and MN841919.

## Abbreviations

AML: Acute myeloid leukemia
BAL: Bronchoalveolar lavage
CLSI: Clinical and Laboratory Standards Institute
CRP: c-reactive protein
CT: Computed tomography
EORTC/MSG: European Organization for Research and Treatment of Cancer/ Mycoses Study Group
EUCAST: European Committee on Antimicrobial Susceptibility Testing
HSCT: Hematopoietic stem cell transplantation
IFI: Invasive fungal infection
ITS: Internal transcribed spacer
lAmB: Liposomal amphotericin B
MIC: Minimum inhibitory concentrations
